# Effects of Lisdexamfetamine, a Prodrug of D-Amphetamine, on Locomotion, Spatial Cognitive Processing and Neurochemical Profiles in Rats: A Comparison With Immediate-Release Amphetamine

**DOI:** 10.3389/fpsyt.2022.885574

**Published:** 2022-04-26

**Authors:** Chen Jian-min, Wang Zhi-yuan, Wu Shi-xuan, Song Rui, Wu Ning, Li Jin

**Affiliations:** Beijing Key Laboratory of Neuropsychopharmacology, State Key Laboratory of Toxicology and Medical Countermeasures, Beijing Institute of Pharmacology and Toxicology, Beijing, China

**Keywords:** lisdexamfetamine, d-amphetamine, spatial cognition, pharmacokinetic characteristics, dopamine

## Abstract

D-amphetamine has been used to enhance cognitive performance over the last few decades. Due to the rapid absorption after administration, d-amphetamine shows narrow effective window and severe abuse potential. Lisdexamfetamine, a prodrug of d-amphetamine, reduces the magnitude of plasma d-amphetamine concentration and prolongs the action duration when compared with immediate-release d-amphetamine at equimolar doses. However, the differences of these two drugs, which produce distinct pharmacokinetic characteristics, in cognition improvement still unclear. In present study, we compared the effects of d-amphetamine (i.p) and lisdexamfetamine (p.o) at equimolar doses (0.2, 0.5, 1.5, 4.5, and 13.5 mg/kg of d-amphetamine base) on locomotion, spatial working memory and recognition memory in rats. Given the crucial involvement of dopamine neurotransmitter system within the medial prefrontal cortex (mPFC) in cognitive processing, microdialysis was conducted to profile the difference in neurochemical characteristics between the two drugs. In our results, d-amphetamine ranges from 0.5 to 1.5 mg/kg significantly increased locomotor activity. However, d-amphetamine ranges from 0.2 to 13.5 mg/kg failed to improve spatial working memory and recognition memory in Y-maze-based spontaneous alternation and two-trial delayed alternation tasks of rats, respectively. In contrast, lisdexamfetamine with 4.5 mg/kg significantly increased the locomotion and improved both spatial working and recognition memory. Further, microdialysis showed that lisdexamfetamine induced lower magnitude and longer duration of extracellular dopamine increase than that of d-amphetamine. These results suggest that lisdexamfetamine was more effective than d-amphetamine in improving spatial cognitive performance, which was attributed to the steady and lasting dopamine release pattern within the mPFC.

## Introduction

Psychostimulants, such as d-amphetamine, modafinil and methylphenidate, have been used to treat attention-deficit/hyperactivity disorder (ADHD) (1, 2), narcolepsy (3) and bipolar disorder (4). In addition, another application of these drugs is to enhance cognition in healthy individuals who are engaging in certain vocations, referred to as cognitive enhancers (5). In the last decades, d-amphetamine has been reported to improve spatial working memory and language production in healthy volunteers (6), as well as increasing vigilance both in boys and adult men (7). However, severe addiction to amphetamine and its analogs (called amphetamine-type stimulants, such as methamphetamine) has become a worldwide public health problem, extensively limiting their applications (8).

Several studies have revealed that both cognitive enhancement and drug addiction are highly associated with pharmacokinetic properties (9, 10). D-amphetamine and methamphetamine enter brain rapidly after administration. They competitively inhibit dopamine transporter (DAT) clearing dopamine (DA), and also release DA via reversing DAT direction, ultimately causing excessive DA accumulation in synaptic cleft ultimately (11). DA acts as an inverted U-shaped pattern to cognitive performance. As is reported, too lower or higher DA level elicited by clinically-inappropriate doses of d-amphetamine impairs cognition (12). In addition, the dramatic increase in DA in the nucleus accumbens (NAc), a key brain region responding to reward (13, 14), is also related to severe abuse and addiction. Thus, it is challenging to change the pharmacokinetic properties of d-amphetamine to increase the effects of cognition improvement, while decrease its potential for abuse.

An alternative strategy is to modify its chemical structure, coupling the active drug with another compound, such as an amino acid, to create a novel prodrug (15). Lisdexamfetamine, the first prodrug approved for ADHD (16, 17) and binge eating disorder (18) treatment, is synthesized by covalently linking d-amphetamine to the amino acid l-lysine (19). Hutson et al. reported that the pharmacodynamic effects of lisdexamfetamine are independent of the route of administration (20), which is enzymatically hydrolyzed by an erythrocyte peptidase (the rate-limiting step) to yield d-amphetamine, the actual pharmacological active metabolite. In comparison with immediate-release d-amphetamine, lisdexamfetamine produced an identical AUC for plasma d -amphetamine, but a 50% lower C_max_ and significantly delayed t_max_ at equimolar doses (21). Such pharmacokinetic profile of lisdexamfetamine shows lower inter- and intra-individual variability in exposure compared with the pharmacokinetic profile of an equivalent dose of immediate-release d-amphetamine (22). Thus, it is reasonable to believe lisdexamfetamine may exhibit wider effective window than d-amphetamine in cognition improvement. Dolder et al. compared the effects of d-amphetamine and lisdexamfetamine on several cognitive tasks in healthy non-sleep-deprived subjects. They just vaguely concluded single, high, equimolar doses of d-amphetamine and lisdexamfetamine enhanced certain aspects of cognitive performance in healthy non-sleep-deprived subjects (14). The exact difference in pharmacological action and mechanisms between d-amphetamine and lisdexamfetamine should be further illumination.

In preclinical study, using appropriate cognitive paradigms in rodents are effective for pharmacological action and the underlying mechanisms exploration, as well as side effects anticipation (23). In fact, several researches have employed various translational rodents paradigms to reflect attention (24), visual discrimination (25) and inhibitory control (26).

Among numerous cognitive domains, working memory serves as the basis of other higher order cognitive processes including but not limited to recognition memory (27–29), which is mediated by mPFC function. In order to compare the effects of d-amphetamine and lisdexamfetamine on mPFC-associating cognition, we focused on the two types of memory: spatial working memory and spatial recognition memory using Y-maze-based spontaneous alternation task (30, 31) and two-trial delayed alteration (32) task respectively. Given that DA plays a vital role in mediating cognitive performance, microdialysis was performed to assess DA, as well as the corresponding metabolites, 3,4-dihydroxyphenylacetic acid (DOPAC) and homovanillic acid (HVA), within the medial prefrontal cortex (mPFC) in freely moving rats to explore the neurochemical profiles of distinct pharmacokinetics induced by d-amphetamine and lisdexamfetamine.

## Materials and Methods

### Animals

Male Sprague-Dawley (SD) rats weighting 220-240 g were purchased from SPF (Beijing) Biotechnology Co., Ltd. (SCXK (Jing) 2019-0010). All rats were housed under a regular light-dark cycle (lights on from 7:00 am to 7:00 pm) at a constant temperature of 22 ± 2°C and relative humidity of 40-60%. The rats were given free access to food and water. The animal protocol was strictly in accordance with the National Institute of Health Guidelines for the Care and Use of Laboratory Animals.

### Drugs and Reagents

D-amphetamine hydrochloride and lisdexamfetamine dimesylate were provided by Beijing Institute of Pharmacology and Toxicology. All drugs were dissolved in sterilized 0.9% saline. D-amphetamine were injected at a volume of 1 ml/kg (intraperitoneal [i.p.]), and lisdexamfetamine was infused at a volume of 2 ml/kg (*per os* [p.o.]).

### Animal Groups and Drug Treatments

SD rats were divided into control, d-amphetamine and lisdexamfetamine treatment groups. Drugs doses were calculated based on free amfetamine base (0.2, 0.5, 1.5, 4.5, 13.5 mg/kg) and transformed to μmol/kg as followed: d-amphetamine: 1.17, 2.91, 8.74, 26.21, 78.64 μmol/kg; lisdexamfetamine: 1.48, 3.70, 11.10, 33.29, 99.86 μmol/kg. For locomotor activity measurement, rats were tested immediately after drug treatment. For the Y-maze spontaneous alternation task, d-amphetamine (i.p.) and lisdexamfetamine (p.o.) were treated 30 and 60 min before the test, respectively. For the two-trial Y-maze delayed alternation task, d-amphetamine (i.p.) and lisdexamfetamine (p.o.) were treated 30 min and 60 min before the first phase (memory acquisition), respectively.

### Locomotor Activity Measurement in Rats

The locomotor activity box was 46 cm × 46 cm × 46 cm and made of black plastic. A camera was fixed to the top of the box. After drug treatment, rats were placed in the box immediately, and locomotion was recorded for 180 min in 15 min interval.

### Y-Maze-Based Spontaneous Alternation in Rats

The apparatus consisted of a Y-shaped maze with three arms (30 cm × 8 cm × 15 cm of each arm, 120° between arms) defined as A, B, and C. Three distinct cues were placed outside the arms to help rats distinguish spatial location. Above the center of the apparatus, a yellow light lamp (1 W) was used to induce a dim environment. All experiments were performed from 8:00 am to 12:00 pm. The procedure was performed as described by Kraeuter et al. (33). Briefly, rats were randomly placed in one of the three arms and allowed to explore freely for 5 min. The maximum alternation is defined as the number of consecutive entries into three different arms (e.g., ABCABC was regarded as four times alteration). Spatial working memory was assessed by the percentage of alterations within 5 min, which can be calculated according to the following formula: number of maximum alternations/(total number of arm entries- 2) × 100. Each rat was placed in a different starting arm.

### Two-Trial Y-Maze-Based Delayed Alternation in Rats

The operation was performed according to the method described by Fu et al. (33, 34). This task consisted of two phases: memory acquisition and memory retrieval. In the first stage, we randomly closed one of the three arms and allowed the rats to freely explore the other two arms for 10 min. After an inter-trial-interval (ITI) of 60 min, the closed arm was opened and the same rat was placed from the same arm as in the first stage and allowed to explore for 5 min. The percentage of novel arm visits (number of novel arm visits/total arm visits × 100) and percentage of novel arm retention (total time in novel arm retention/total time in three arms × 100) within 5 min were calculated to reflect the spatial recognition memory of rats.

### Surgery

Briefly, rats were anesthetized with pentobarbital sodium (50 mg/kg, i.p.). The head was placed in a stereotaxic apparatus. The upper incisor bar was set at 3.3 mm below the interaural line so that the skull surface between the bregma and lambda was horizontal according to Paxinos and Watson (35). A microdialysis guide cannula (CMA, United Kingdom) was implanted at the following coordinates: AP, 3.0 mm; ML, 0.6 mm relative to bregma; and V, 2.5 mm relative to skull surface. In addition, two additional burr holes were made for skull screws (stainless steel) and secured using dental cement. After surgery, rats were injected with benzylpenicillin sodium (300,000 IU/kg, i.p.) to prevent infection.

### Microdialysis and High-Performance Liquid Chromatography-Electrochemical Detection (HPLC-ECD) Analysis

Following 5-7 days of recovery after surgery, the microdialysis experiment was conducted. A microdialysis probe (EICOM A-I: 0.22-mm OD, 4-mm membrane length with 50 kDa cutoff) was inserted into the guide cannula of awake rats. The rats were then perfused with artificial cerebrospinal fluid (aCSF; containing 148 mM NaCl, 4 mM KCl, 0.8 mM MgCl_2_, 1.4 mM CaCl_2_, 1.2 mM Na_2_HPO_4_, 0.3 mM NaH_2_PO_4_, pH 7.2) at a low speed (0.5 μl/min) overnight. On the next day, the perfusion speed was increased to 1.0 μl/min for 2 h. The dialysate samples were collected at 30-min intervals from 90 min before drug administration to 3 h after drug administration. The collection vials contained 7.5 μl of 0.5 M perchloric acid to prevent oxidation of catecholamines.

Reverse-phase, ion-pair HPLC (Waters 2695, MA, United States) coupled with ECD (Antect Leyden, Zoeterwoude, and Holland) was used to analyze DA and the corresponding metabolites DOPAC and HVA. The collected samples were immediately analyzed. Samples (30 μl) were separated using a 250 × 4.5-mm IDT3 analytical column (Waters) at 1.0 ml/min. The mobile phase consisted of 100 mM phosphate buffer, 0.74 mM sodium 1-octanesulfonate, 0.027 mM EDTA⋅Na_2_ at pH 3.0, 8% (v/v) methanol, and 8% (v/v) acetonitrile; an Antec Intro ECD was used with a high-density, glassy carbon electrode (+ 0.72 V) combined with an Ag/AgCl reference electrode.

### Nissl Staining

After dialysis, brain tissues were stained with cresyl violet to detect the surgery position. Briefly, rats were anesthetized with pentobarbital sodium (50 mg/kg, i.p.) and perfused with 0.9% saline and 4% paraformaldehyde to fix the brain tissue. After that, the brain was immersed in 4% paraformaldehyde for 24 h, and dehydrated with 30% sucrose for 24 h. Brain tissue was embedded in O.C.T compound (SAKURA) and sectioned into 30 μm-thick sections using a freezing microtome (Leica, Germany). The Nissl staining protocol was performed according to the manufacturer’s instructions. Images were captured using a NanoZoomer Digital Pathology microscope (Hamamatsu Photonics, Japan). The injection site of the guide cannula, which was not in the mPFC, was deleted.

### Statistical Analysis

Levene’s homogeneous variance test and Shapiro-Wilk normal test were used to analysis the homogeneous variance and normal distribution of (1) total distance traveled over 180 min, (2) percentage of alterations within 5 min in the Y-maze-based spontaneous alternation, and (3) percentage of novel arm visits and retention in the two-trial Y-maze-based delayed alternation. Data was presented as mean ± SEM when conform to homogeneous variance and normal distribution, and one-way analysis of variance (ANOVA) followed by Dunnett’s t-test was used for analysis. Otherwise, data were presented as median ± interquartile and Kruskal-Wallis test was employed for analysis. For the 15 min interval distances within 180 min in locomotor activity experiment and the percentage of each arm visits and retention in two-trial Y-maze-based delayed alternation experiment (treatment as between-group factor, arm differences as within-group factor), data were presented as mean ± SEM and two-way ANOVA with one repeated measurement followed by Bonferroni test was performed. All statistical analyses were performed using SPSS software (version 26.0). Statistical significance was set at *P* < 0.05.

## Results

### Effects of D-Amphetamine and Lisdexamfetamine on Locomotor Activity in Rats

**FIGURE 1:**
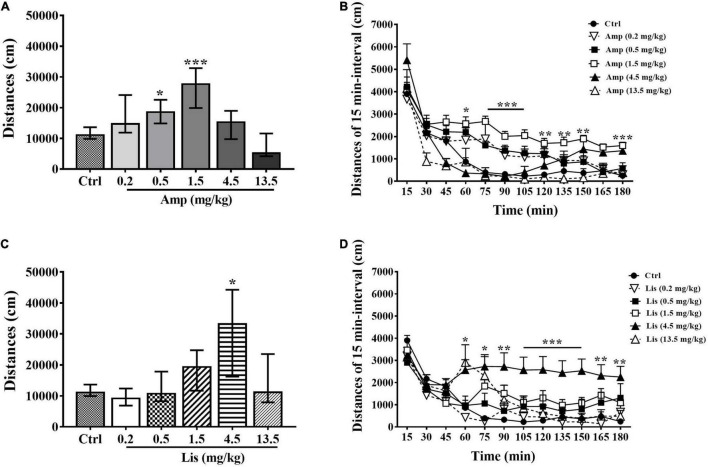
The effects of d-amphetamine and lisdexamfetamine on locomotor activity. **(A)** Total distances induced by d-amphetamine within 180 min (median ± interquartile, Kruskal-Wallis test). **(B)** Distances induced by d-amphetamine with 15-min interval (mean ± SEM, Repeated measure ANOVA followed by Bonferroni test). **(C)** Total distances induced by lisdexamfetamine within 180 min (median ± interquartile, Kruskal-Wallis test). **(D)** Distances induced by lisdexamfetamine with 15-min interval (mean ± SEM, Repeated measure ANOVA followed by Bonferroni test). Ctrl: control; Amp: d-amphetamine; Lis: lisdexamfetamine. *P < 0.05, **P < 0.01, ***P < 0.001 *vs*. Ctrl, *n* = 9 in each group.

Generally, d-amphetamine increased locomotor activity of rats at dosage of 0.2-1.5 mg/kg, whereas decreased locomotor activity at dosage of 4.5-13.5 mg/kg. In comparison with the control group, 0.5 and 1.5 mg/kg d-amphetamine significantly enhanced locomotor activity over a total of 180 min (Kruskal-Wallis test: *P* = 0.018 for 0.5 mg/kg and *P* < 0.001 for 1.5 mg/kg *vs.* control, Figure 1A). The results for the 15-min interval distances showed that 1.5 mg/kg produced the lasting increased locomotion from 60 to 180 min after administration (two-way ANOVA with one repeated measurement: main effect of time: F_(11,38)_ = 25.818, *P* < 0.001; main effect of treatment: F_(5,48)_ = 10.391, *P* < 0.001; time × treatment interaction: F_(55,210)_ = 1.519, *P* = 0.019; Bonferroni test for *post hoc* test: *P* < 0.05 from 60 min to 180 min *vs*. control, Figure 1B).

Lisdexamfetamine exhibited an inverted-U-shaped dose-response relationship with locomotion. Specifically, 4.5 mg/kg lisdexamfetamine significantly increased locomotion within 180 min (Kruskal-Wallis test: *P* = 0.016 *vs.* control, Figure 1C). The results for the 15-min interval distances showed that 4.5 mg/kg lisdexamfetamine significantly increased locomotor activity from 75 min to 180 min, while a dose of 13.5 mg/kg significantly increased activity at 60 min after administration (two-way ANOVA with one repeated measurement: main effect of time: F_(11,38)_ = 21.488, *P* < 0.001 main effect of treatment: F_(5,48)_ = 4.899, *P* = 0.001; time × treatment interaction: F_(55,210)_ = 1.594, *P* = 0.01; Bonferroni test for *post hoc* test: 4.5 mg/kg: *P* < 0.05 from 75 to 180 min, 13.5 mg/kg: *P* = 0.027 at 60 min *vs.* control, Figure 1D].

### Effects of D-Amphetamine and Lisdexamfetamine on Y-Maze-Based Spontaneous Alternation in Rats

**FIGURE 2:**
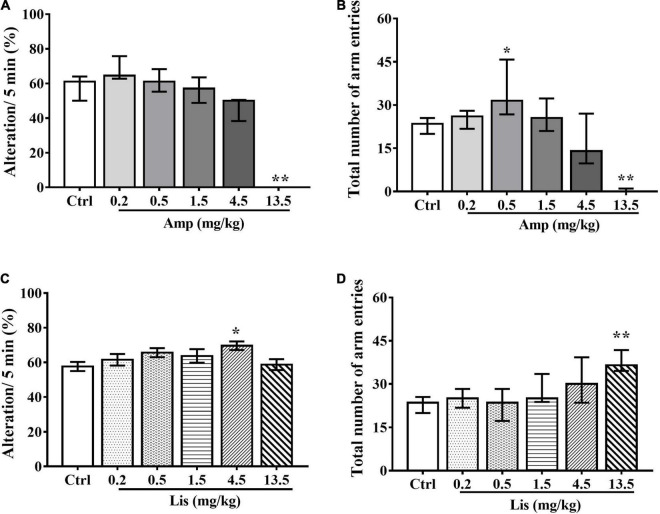
The effects of d-amphetamine and lisdexamfetamine on Y-maze-based spontaneous alternation. **(A)** The effect of d-amphetamine on percentage of Alteration/5min (median ± interquartile, Kruskal-Wallis test). **(B)** The effect of d-amphetamine on transform times within 5 min (median ± interquartile, Kruskal-Wallis test). **(C)** The effect of lisdexamfetamine on percentage of Alteration/5 min (mean ± SEM, One-way ANOVA, followed by Dunnett-*t* test). **(D)** The effect of lisdexamfetamine on transform times within 5 min (median ± interquartile, Kruskal-Wallis test). Ctrl: control; Amp: d-amphetamine; Lis: lisdexamfetamine. *P < 0.05, **P < 0.01 vs. Ctrl, *n* = 10 in each group.

In rats, as d-amphetamine dose increased ranging from 0.2-13.5 mg/kg, the percentage of spontaneous alterations within 5 min was gradually decreased. Compared with the control group, 13.5 mg/kg d-amphetamine significantly reduced spontaneous alternations (Kruskal-Wallis test: *P* = 0.004 *vs.* control, Figure 2A). In total number of arm entries, d-amphetamine at dosage of 0.5 mg/kg significantly increased, whereas 13.5 mg/kg d-amphetamine significantly reduced arm visiting times (Kruskal-Wallis test: *P* = 0.048 for 0.5 mg/kg and *P* = 0.001 for 13.5 mg/kg *vs.* control, Figure 2B).

Compared with control group, lisdexamfetamine at dosage of 4.5 mg/kg significantly increased the percentage of spontaneous alterations within 5 min in rats (One-way ANOVA followed by Dunnett’s *t*-test: *P* = 0.032 *vs.* control, Figure 2C). Total number of arm entries were significantly increased by 13.5 mg/kg lisdexamfetamine (Kruskal-Wallis test: *P* < 0.001 *vs.* control, Figure 2D).

### Effects of D-Amphetamine and Lisdexamfetamine on Y-Maze-Based Delayed Alternation in Rats

**FIGURE 3:**
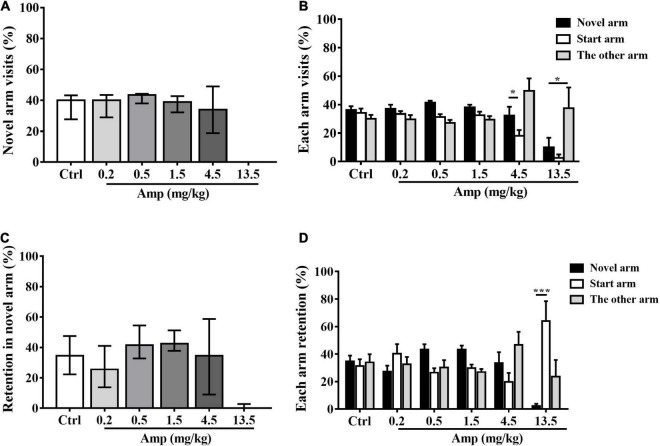
The effects of d-amphetamine on Tow-trial Y-maze based delayed alternation. **(A)** The effect of d-amphetamine on percentage of novel arm visit times (median ± interquartile, Kruskal-Wallis test). **(B)** The percentage of each arm visits (mean ± SEM, Repeated measure ANOVA followed by Bonferroni test). **(C)** The effect of d-amphetamine on percentage of novel arm retention (median ± interquartile, Kruskal-Wallis test). **(D)** The percentage of each arm retention (mean ± SEM, Repeated measure ANOVA followed by Bonferroni test). Ctrl: control; Amp: d-amphetamine. **(A,C)** *P < 0.05, ***P < 0.001 vs. Ctrl; **(B,D)** *P < 0.05, ***P < 0.001 vs. Novel arm, *n* = 10 in each group.

Generally, d-amphetamine ranges from 0.2 to 13.5 mg/kg increased both the percentage of novel arm visits and retention at lower doses and then decreased at higher doses in rats. Specifically, 13.5 mg/kg led to a significant reduction in the percentage of retention in the novel arm compared with the control group (Kruskal-Wallis test: *P* = 0.001 in 13.5 mg/kg *vs.* control, Figures 3A,C). Two-way ANOVA with one repeated measurement revealed that 4.5 mg/kg d-amphetamine produced more novel arm visits than start arm visits, while 13.5 mg/kg caused fewer novel arm visits than start and other arm visits (**arm visits:** main effect of treatment: F_(5,54)_ = 8.995, *P* < 0.001; main effect of arm: F_(2,53)_ = 9.489, *P* < 0.001, treatment × arm interaction: F_(10,108)_ = 2.543, *P* = 0.011; Bonferroni test for *post hoc* test: 4.5 mg/kg: *P* = 0.046 in novel arm *vs.* start arm, 13.5 mg/kg: *P* = 0.017 in novel arm *vs.* the other arm. **arm retention:** main effect of treatment: F_(5,54)_ = 0.991, *P* = 0.432; main effect of arm: F_(2,53)_ = 0.714, *P* = 0.494; treatment × arm interaction: F_(10,108)_ = 4.347, *P* < 0.001, Figures 3B,D).

**FIGURE 4:**
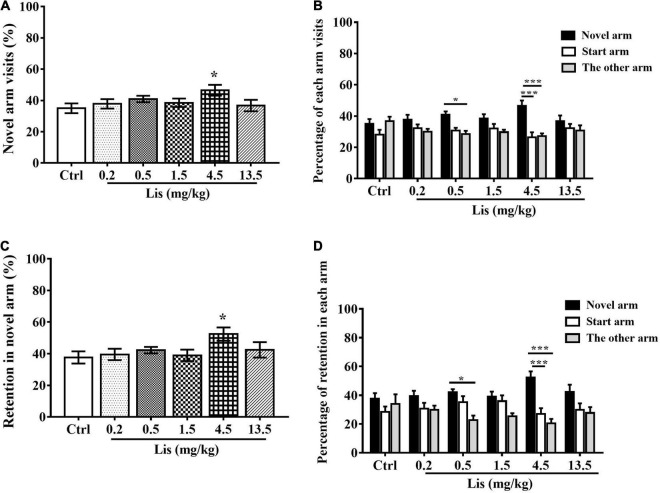
The effects of lisdexamfetamine on Tow-trial Y-maze based delayed alternation. **(A)** The effect of lisdexamfetamine on percentage of novel arm visit times (mean ± SEM, One-way ANOVA, followed by Dunnett-*t* test). **(B)** The percentage of each arm visits (mean ± SEM, Repeated measure ANOVA followed by Bonferroni test). **(C)** The effect of lisdexamfetamine on percentage of novel arm retention (mean ± SEM, One-way ANOVA, followed by Dunnett-*t* test). **(D)** The percentage of each arm retention (mean ± SEM, Repeated measure ANOVA followed by Bonferroni test). Ctrl: control; Caf: caffeine; Lis: lisdexamfetamine. **(A,C)** *P < 0.05, **P < 0.01, ***P < 0.001 *vs*. Ctrl; **(B,D)** *P < 0.05, **P < 0.01, ***P < 0.001 *vs*. novel arm, *n* = 10 in each group.

In comparison with the control group, 4.5 mg/kg lisdexamfetamine significantly increased both the percentage of novel arm visits (one-way ANOVA followed by Dunnett’s t-test: *P* = 0.047 *vs.* control, Figure 4A) and the percentage of retention in the novel arm (one-way ANOVA followed by Dunnett’s t-test: *P* = 0.043 *vs.* control, Figure 4C). Two-way ANOVA with one repeated measurement revealed that 0.5 and 4.5 mg/kg lisdexamfetamine significantly increased the number of visits and retention in the novel arm relative to those in the start and other arm (**arm visits:** treatment main effect: F_(5,54)_ = 1.413, *P* = 0.234, arm main effect: F_(2,53)_ = 13.995, *P* < 0.001, treatment × arm interaction: F_(10,108)_ = 1.489, *P* = 0.153, Bonferroni test for *post hoc* test: 0.5 mg/kg: *P* = 0.032 in novel arm *vs.* the other arm, 4.5 mg/kg: *P* < 0.001 in novel arm *vs.* start arm and other arm; **arm retention:** treatment main effect: F_(6_,_63)_ = 0.849, *P* = 0.537; arm main effect: F_(2,53)_ = 16.257, *P* < 0.001; treatment × arm: F_(10,108)_ = 1.421, *P* = 0.181; Bonferroni test for *post hoc* test: 0.5 mg/kg: *P* = 0.013 in novel arm *vs.* the other arm, 4.5 mg/kg: *P* < 0.001 in novel arm *vs.* start arm and the other arm, Figures 4B,D).

### Effects of D-Amphetamine and Lisdexamfetamine on mPFC DA, DOPAC, and HVA Levels in Rats

**FIGURE 5:**
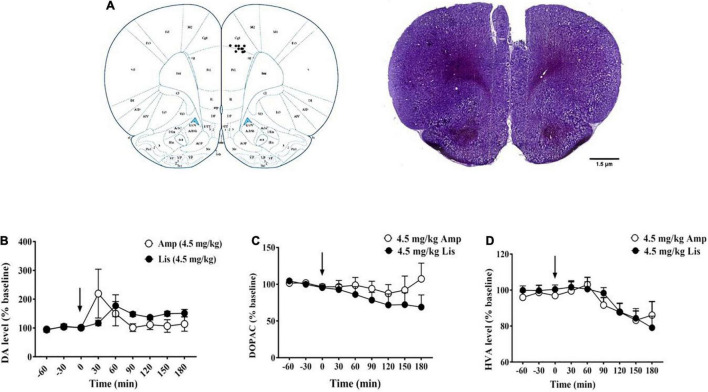
The effects of d-amphetamine and lisdexamfetamine on catecholamine neurotransmitters and the metabolites in mPFC. **(A)** The implantation location of the microdialysis guide cannula. **(B)** The effects of d-amphetamine and lisdexamfetamine on DA efflux. **(C)** The effects of d-amphetamine and lisdexamfetamine on DOPAC efflux. **(D)** The effects of d-amphetamine and lisdexamfetamine on HVA efflux. Amp: d-amphetamine; Lis: lisdexamfetamine. Data are presented by mean ± SEM, *n* = 5 in each group.

Figure 5A showed the implantation location of the microdialysis guide cannula. For DA, d-amphetamine dramatically increased DA efflux (% of baseline: 222.49 ± 84.42) at 30 min after administration, whereas lisdexamfetamine induced extracellular DA elevation and peaked (% of baseline: 177.35 ± 37.94) at 60 min after administration. As time passed, the increased DA levels induced by d-amphetamine gradually returned to baseline from 90 min after administration (% of baseline: 90 min: 108.25 ± 16.28, 120 min: 112.36 ± 17.36, 150 min: 100.31 ± 21.41, 180 min: 104.02 ± 24.13). However, lisdexamfetamine had a lower magnitude effect and longer duration of DA efflux from 90 to 180 min (% of baseline: 90 min: 148.07 ± 7.85, 120 min: 136.88 ± 4.98, 150 min: 149.73 ± 8.49, 180 min: 151.31 ± 13.27, Figure 5B). For DOPAC, there were obvious differences between d-amphetamine and lisdexamfetamine. D-amphetamine led to a gradual decrease in DOPAC concentration from 30 to 120 min, reaching its lowest (% baseline: 87.52 ± 12.04) at 120 min after administration, and then returning to approximately the baseline level. However, lisdexamfetamine led to a constant decrease in DOPAC concentration, reaching the lowest levels at 180 min (% baseline: 68.95 ± 16.61, Figure 5C). For HVA, there was no difference between d-amphetamine and lisdexamfetamine, which both led to a gradual decrease in HVA concentration (Figure 5D).

## Discussion

D-amphetamine, due to the immediate-released pharmacokinetic characteristics, shows narrow effective window in cognition improvement. Lisdexamfetamine, based on the sustained-released pharmacokinetic profile with lower magnitude of plasma d-amphetamine concentration, is reasonably believed to produce wider efficient and less individual variability to enhance cognitive performance. The present study found that lisdexamfetamine was more effective than immediate-released d-amphetamine in improving spatial cognitive performance in rats, which was attributed to its inducing the steady and lasting dopamine release pattern within the mPFC.

Locomotor activity measurement is a method used to evaluate the behavioral stimulant properties of drugs (36). To further study the pharmacological actions on cognitive performance, it is necessary to firstly explore the neuronal excitatory effects of lisdexamfetamine and d-amphetamine. In our results, 0.5 and 1.5 mg/kg d-amphetamine significantly increased locomotor activity after i.p. administration, revealing neuronal stimulation caused by this dose. Further increased dose to 13.5 mg/kg, d-amphetamine significantly decreased locomotion 30 min after administration, which is similar to findings in other studies. Namara et al. found 0.2 mg/kg d-amphetamine (i.p.) failed to increase locomotor activity, while doses of 5 and 10 mg/kg d-amphetamine caused prominent stereotypy (37). In addition, Antoniou et al. concluded that amphetamine had a complex effect on locomotion, characterized mainly by motor activation at lower doses and stereotypy at high doses (36). Unlike d-amphetamine, lisdexamfetamine from 4.5 to 13.5 mg/kg increased locomotor activity from 60 min to 90 min after administration (p.o.). These results suggest that lisdexamfetamine produced substantially less locomotor activation than d-amphetamine due to its sustained and lower magnitude pharmacokinetic profile. Rowley et al. (21) also reported that lisdexamfetamine at 1.5 mg/kg produced less locomotor activation than that of equivalent dose of d-amphetamine, which is similar to our results. Due to the unique pharmacokinetics, lisdexamfetamine shows a lower reward property and larger therapeutic window than d-amphetamine (21, 38).

Increasing neuronal excitability appropriately with a certain dose of stimulant drugs is beneficial for enhancing cognitive activity. Based on the locomotor activity measure, we compared the stimulant properties of lisdexamfetamine and d-amphetamine, and found that lisdexamfetamine was less stimulating than d-amphetamine. However, the effects of both drugs on cognitive performance remain unknown. As doses increased to levels that stimulated locomotion (0.5-1.5 mg/kg), d-amphetamine failed to improve working memory; further increases to 13.5 mg/kg significantly reduced spatial working memory, which may associate with stereotype behavior. In fact, psychostimulants (i.e., d-amphetamine) action on the DA system exerts bidirectional effects, improving or decreasing working memory performance depending on the dosage (inverted U-shaped curve) (39, 40). It is widely accepted that clinically relevant doses improve and supra-clinical doses impair working memory (41). This standpoint has also been confirmed by other studies. Brut et al. found that acute treatment with low doses of d-amphetamine (1.0 mg/kg) eliminated the alternation tendency, while higher doses (5.0- 7.0 mg/kg) also produced marked stimulus perseveration in a radial maze (42). In addition, several lines of evidence have shown that d-amphetamine causes significantly perseverative patterns (i.e., repetition of location rather than direction) in exploration in the Y-maze (43, 44). Except for dosage, baseline working memory capacity is another key factor affecting cognitive performance. D-amphetamine selectively improved working memory in poor and intermediate performers at low doses, whereas it was impaired good performers at a higher dose (14, 45). In our animal results and other studies of healthy non-sleep-deprived individuals, d-amphetamine failed to improve spatial working memory (46). D-amphetamine exhibits a narrow effective window for cognitive improvement. Unlike d-amphetamine, lisdexamfetamine (4.5 mg/kg) significantly improved working memory. As the dose increased to 13.5 mg/kg, lisdexamfetamine did not significantly impair performance. To our knowledge, only one study has reported that chronic lisdexamfetamine treatment effectively enhanced spatial working memory in the Morris water maze (15), which is consistence to our results. In a word, our results suggested the reduced rate of appearance and magnitude of d-amphetamine in plasma by lisdexamfetamine may be more beneficial in working memory improvement.

The two-trial Y-maze delayed alternation task is a specific and sensitive test of spatial recognition memory in rodents (47, 48). This paradigm requires rats to explore and remember two arms first (memory acquisition). After a 60-min ITI, rats spend more time in the novel arm because of their natural exploration tendency (memory expression). Chronic administration and withdrawal of amphetamine and morphine have been shown to cause severe spatial recognition memory and executive impairment in rodents and humans (49–51). However, whether acute administration of amphetamine influences this cognitive domain and whether there is a difference between amphetamine and lisdexamfetamine remain unknown. In our study, d-amphetamine was administered before the first training phase, affecting memory acquisition. Our results showed that acute treatment with 4.5 and 13.5 mg/kg d-amphetamine significantly damaged recognition memory acquisition and showed similar effects on spatial working memory. Exploration between two given arms is crucial to guide free exploration after a 60-min ITI. Thus, larger doses of d-amphetamine may cause perseverative behavior in the first exploration, causing rats to fail to remember and distinguish which arm is the novel one. Compared to d-amphetamine, lisdexamfetamine (4.5 mg/kg significantly improved spatial recognition memory acquisition, upon further increasing the dose to 13.5 mg/kg, lisdexamfetamine did not produce a significant impairment relative to that of d-amphetamine. The results here were consistent to that of Y-maze-based spontaneous alternation.

DA within the mPFC plays a crucial role in mediating several cognitive domains, such as working memory (52) and attention (53). We demonstrated that lisdexamfetamine (4.5 mg/kg d-amphetamine base) significantly enhanced spatial working memory and spatial recognition memory in comparison to equivalent doses of d-amphetamine. Thus, we speculated that the neurochemical profiles of lisdexamfetamine and d-amphetamine are different, so that the release pattern elicited by lisdexamfetamine may be more beneficial to improving cognitive performance. Here, we found that lisdexamfetamine evoked a smaller magnitude but sustained increase in DA levels within the mPFC compared to that with d-amphetamine (222% of baseline for d-amphetamine at 30 min and 177% for lisdexamfetamine at 60 min after administration), which is similar to the case in the striatum and the pharmacokinetic characteristics reported by Rowley et al. (21). Except for DA, we also observed that both stimulants decreased DOPAC and HVA levels. D-amphetamine produced a transient decrease in extracellular DOPAC levels, which is consistent with several other results (54, 55). DOPAC is a major metabolite of DA, catalyzed by monoamine oxidase (MAO), and further forms HVA under the action of catechol-o-methyl-transferase (COMT). D-amphetamine has been proven to decrease MAO activity in striatal tissue (56), contributing to a reduction in DOPAC and final metabolite HVA reduction.

Taken together, as a prodrug of d-amphetamine, lisdexamfetamine displays pharmacokinetical sustained and lower magnitude plasma amphetamine base concentration, as well as its eliciting DA level in mPFC. This unique characteristic may be more benefit to improve cognition than immediate-release d-amphetamine.

## Data Availability Statement

The raw data supporting the conclusions of this article will be made available by the authors, without undue reservation.

## Ethics Statement

The animal study was reviewed and approved by National Institute of Health Guidelines for the Care and Use of Laboratory Animals.

## Author Contributions

CJ-M and WS-X performed the behavioral experiments. CJ-M and WZ-Y analyzed the data. SR conducted *in vivo* microdialysis experiment. CJ-M wrote the manuscript. WN and LJ designed the experiments and modified the manuscript. All authors contributed to the article and approved the submitted version.

## Conflict of Interest

The authors declare that the research was conducted in the absence of any commercial or financial relationships that could be construed as a potential conflict of interest.

## Publisher’s Note

All claims expressed in this article are solely those of the authors and do not necessarily represent those of their affiliated organizations, or those of the publisher, the editors and the reviewers. Any product that may be evaluated in this article, or claim that may be made by its manufacturer, is not guaranteed or endorsed by the publisher.
